# Proposed Regulation of Gene Expression by Glucose in Rodent Heart

**DOI:** 10.4137/grsb.s222

**Published:** 2007-11-05

**Authors:** Martin E. Young, Jie Yan, Peter Razeghi, Robert C. Cooksey, Patrick H. Guthrie, Stanislaw M. Stepkowski, Donald A. McClain, Rong Tian, Heinrich Taegtmeyer

**Affiliations:** 1 USDA/ARS Children’s Nutrition Research Center, Baylor College of Medicine, Houston, Texas; 2 Division of Cardiovascular Medicine, Brigham and Women’s Hospital and Harvard Medical School, Boston, Massachusetts; 3 Division of Cardiology, The University of Texas-Houston Medical School, Houston, Texas; 4 Division of Endocrinology, Metabolism and Diabetes, University of Utah, Salt Lake City, Utah; 5 Division of Organ Transplantation, Department of Surgery, The University of Texas-Houston Medical School, Houston, Texas

**Keywords:** Heart, Glucose Metabolism, Fetal Gene Program

## Abstract

**Background:**

During pressure overload-induced hypertrophy, unloading-induced atrophy, and diabetes mellitus, the heart induces ‘fetal’ genes (e.g. myosin heavy chain β; *mhcβ*).

**Hypothesis:**

We propose that altered glucose homeostasis within the cardiomyocyte acts as a central mechanism for the regulation of gene expression in response to environmental stresses. The evidence is as follows.

**Methods and Results:**

Forced glucose uptake both *ex vivo* and *in vivo* results in *mhc* isoform switching. Restricting dietary glucose prevents *mhc* isoform switching in hearts of both GLUT1-Tg mice and rats subjected to pressure overload-induced hypertrophy. Thus, glucose availability correlates with *mhc* isoform switching under all conditions investigated. A potential mechanism by which glucose affects gene expression is through O-linked glycosylation of specific transcription factors. Glutamine:fructose-6-phosphate amidotransferase (GFAT) catalyzes the flux generating step in UDP-N-acetylglucosamine biosynthesis, the rate determining metabolite in protein glycosylation. Ascending aortic constriction increased intracellular levels of UDP-N-acetylglucosamine, and the expression of *gfat2*, but not *gfat1*, in the rat heart.

**Conclusions:**

Collectively, the results strongly suggest glucose-regulated gene expression in the heart, and the involvement of glucose metabolites in isoform switching of sarcomeric proteins characteristic for the fetal gene program.

## Introduction

The heart adapts to changes in its physiologic environment, initially through alterations in the activity state and/or locality of pre-existing proteins within the cardiomyocyte. Chronic activation of intracellular signaling cascades affect the levels of specific proteins in the cell, through transcriptional, translational and post-translational mechanisms. For example, increased workload acutely increases glycogenolysis, glucose transport, and lactate oxidation, with a lesser effect on fatty acid oxidation (Goodwin et al. 1998). Persistence of pressure overload on the heart results in sustained alterations in metabolism as a consequence of changes in the expression of proteins involved in glucose and fatty acid utilization (Allard et al. 1994).

Sustained pressure overload not only affects substrate preference, but also leads to a remodeling of the heart. Cardiomyocytes increase in size (hypertrophy), together with alterations in the expression of multiple myocardial genes (Kleinman et al. 1978; Sugden and Clerk, 1998). In the latter case, the hypertrophied heart reverts to a fetal pattern of gene expression, wherein various fetal genes are induced with a concomitant repression of adult genes (Komuro and Yazaki, 1993; Depre et al. 1998). One example is the switching of *mhc* isoforms, in which *mhcβ* (the fetal isoform) is induced, while *mhcα* (the adult isoform) is repressed in the hypertrophied rat heart (Depre et al. 1998; Mercadier et al. 1981). The exact mechanism(s) by which this isoform switching occurs is (are) not known. It is an intriguing observation that sustained decreases in work load or sustained changes in the metabolic milieu (e.g. insulin deficient diabetes mellitus) induce a similar patter of isoform switching (Depre et al. 1998; Dillmann, 1980; Depre et al. 2000).

The purpose of the present study was to explore the hypothesis that substrate metabolism and/or intracellular metabolites are responsible for *mhc* isoform switching in the heart in response to stimuli such as pressure overload. More specifically, we hypothesized that accumulation of intramyocellular glucose metabolites drives *mhc* isoform switching in the hypertrophied heart. This hypothesis was investigated by: 1) increasing glucose influx into the heart *ex vivo*; 2) increasing glucose influx into the heart *in vivo*; 3) attenuating increased glucose influx by the heart during pressure overload-induced hypertrophy *in vivo*; and 4) investigating potential glucose sensing components in both the normal and pressure overloaded heart. The results are consistent with the notion that alterations in glucose metabolism play a role in *mhc* isoform switching.

## Methods

### Animals and dietary interventions

Rats: Male Spraque-Dawley rats were kept in the Animal Care Center of the University of Texas-Houston Medical School. Mice: Male wild-type and MHCα-GLUT1 transgenic littermates with cardiac-specific overexpression of GLUT1 were kept in the Animal Resource Facility at Harvard Medical School. All rodents were housed under controlled conditions (23 ± 1 °C; 12 h light/12 h dark cycle) and received chow and water *ad libitum*. The investigations conformed to the *Guide for the Care and Use of Laboratory Animals* published by the US National Institutes of Health (NIH Publication No. 85-23, revised 1996).

Rodents were fed one of three isocaloric diets; standard laboratory chow (Purina), a high carbohydrate/low fat diet (HC/LF; Research Diets Inc., New Brunswick, NJ), or a low carbohydrate/high fat diet (LC/HF; Research Diets Inc., New Brunswick, NJ). Because the latter two diets were isocaloric, they varied only in the proportion of energy obtained from carbohydrate and fat. For the rat studies, the contribution of carbohydrate, fat and protein to total energy available were 71%, 6% and 23% for the HC/LF diet, and 24%, 53% and 23% for the LC/HF diet, respectively. For the mouse studies, the contribution of carbohydrate, fat and protein to total energy available were 70%, 10% and 20% for the HC/LF diet, and 20%, 60% and 20% for the LC/HF diet, respectively. The fat component consisted chiefly of lard (saturated, long-chain fatty acids). All other animals were fed the standard laboratory chow.

### Working heart *ex vivo*

Isolated rat hearts were perfused for 90 min in the working mode (15 cm H_2_O preload/100 cm H_2_O afterload) with Krebs-Henseleit buffer containing β-hydroxybutyrate (10 mM), acetoacetate (1 mM) and propionyl-L-carnitine (2 mM). In certain experiments, glucose (25 mM) was added to the perfusate. Mannitol (25 mM) was added to the perfusate when glucose was absent. A physiologic concentration of insulin (40 μU/ml; Lilly) was included in all perfusions. At the end, hearts were freeze-clamped and stored in liquid nitrogen. This method has been described in detail elsewhere (Taegtmeyer et al. 1980).

### Ascending aortic constriction *in vivo*

Cardiac pressure overload was induced by constriction of the ascending aorta (Kleinman et al. 1978). In control animals, sham operations were performed. During the seven days before surgery, the animals received either standard laboratory chow (control diet), the HC/LF diet, or the LC/HF diet. Specific feeding was maintained until the completion of the experiment (i.e. isolation of hearts), either nine days (HC/LF and LC/HF feeding studies) or seven weeks (standard laboratory chow studies) after surgery. The effects of banding and altered dietary fat content on body weight and heart weight are shown in Table 1.

### RNA isolation and quantitative RT-PCR

RNA extraction and quantitative RT-PCR of samples was performed using previously described methods (Depre et al. 1998; Chomczynski and Sacchi 1987; Gibson et al. 1996). Specific quantitative assays were designed for *mhcα*, *mhcβ*, *gfat1*, *gfat2*, glucose 6-phosphate dehydrogenase (*g6pdh*), and transketolase (*tkl)* using rat and mouse sequences available in GenBank (Table 2). Primers and probes were designed from non-conserved sequences of the genes (allowing for isoform specificity), spanning sites where two exons join (splice sites), preventing recognition of the assay to any potential contaminating genomic DNA. Standard RNA was made for all assays by the T7 polymerase method (Ambion, Austin, Texas), using total RNA isolated from rat hearts. The correlation between the C_t_ (the number of PCR cycles required for the fluorescent signal to reach a detection threshold) and the amount of standard was linear over at least a 5-log range of RNA for all assays (data not shown). PCR data are reported as the number of mRNA transcripts per ng total RNA.

### UDP-N-Acetylglucosamine

The levels of UDP-N-acetylglucosamine, the major end product of the hexosamine biosynthetic pathway, were measured in perchloric acid extracts of rat heart homogenates using high-performance liquid chromatography and spectrophotometry as previously described (Veerababu et al. 2000).

### Xylulose 5-Phosphate

Myocardial levels of the pentose phosphate pathway (PPP) intermediate xylulose 5-phosphate were measured using a spectrophotometric assay similar to that described previously for the liver (Casazza and Veech, 1986). Briefly, deproteinized heart samples were prepared by homogenizing approximately 400 mg of powdered heart with 5.6% perchloric acid (2 mls). Following neutrilization, xylulose 5-phosphate levels were measured in PCA extracts by following NADH generation via the combined transketolase and glyceraldehyde 3-phosphate dehydrogenase reactions.

### Statistical analysis

Data are presented as the mean ± SEM for between four and ten hearts in each group. Statistically significant differences between groups were calculated by analysis of variance (ANOVA). A value of p < 0.05 was considered significant.

## Results

### Glucose induces *mhc* isoform switching *ex vivo*

**Figure 1:**
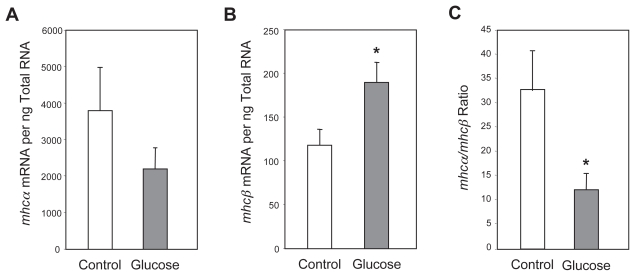
Effects of glucose on *mhcα* (**A**) and *mhcβ* (**B**) expression, as well as the *mhcα*/*mhcβ* ratio (**C**), in the perfused working rat heart. Hearts were perfused for 90 minutes in Krebs-Henseleit buffer containing β-hydroxybutyrate (10 mM), acetoacetate (1 mM), propionyl-L-carnitine (2 mM) and insulin (40 μUnits/ml), without (control) or with glucose (25 mM). Control hearts were also perfused in the presence of mannitol (25 mM). Values are shown as the mean ±S.E.M. for four separate observations. *, p < 0.05.

To determine whether glucose induced *mhc* isoform switching in hearts under controlled conditions *ex vivo*, the isolated working rat heart preparation was utilized. Working rat hearts from animals fed a standard laboratory chow diet were perfused either in the absence or presence of glucose (25 mM). In the former case, mannitol (25 mM) was added to the perfusate to allow for any osmotic effects of glucose on heart function and/or gene expression. A trend for lower levels of *mhcα* mRNA was observed for hearts perfused in the presence of glucose compared to those perfused in the presence of mannitol, although this did not reach statistical significance (Fig. 1A). However, *mhcβ* mRNA levels were significantly elevated in hearts perfused with glucose, compared to those perfused with mannitol (Fig. 1B). The *mhcα*/*mhcβ* ratio (a marker of isoform switching) was significantly lower in glucose-perfused hearts (Fig. 1C).

### Glucose induces *mhc* isoform switching *in vivo*

**Figure 2:**
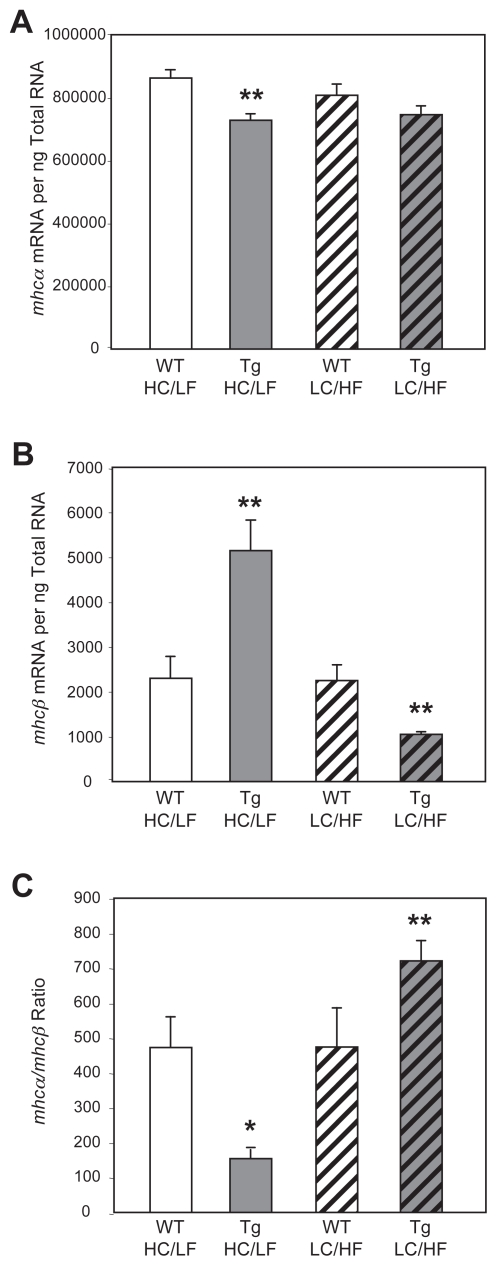
Effects of altered dietary carbohydrate and fat availability on *mhcα* (**A**) and *mhcβ* (**B**) expression, as well as the *mhcα*/*mhcβ* ratio (**C**), in wild-type (WT) and GLUT1 transgenic (Tg) hearts. Mice were placed on either a high carbohydrate/low fat diet (HC/LF) or a low carbohydrate/high fat diet (LC/HF). Values are shown as the mean ± S.E.M. for six separate observations. *, p < 0.05, and **, p < 0.01 *versus* WT.

To investigate whether glucose induced *mhc* isoform switching occurs *in vivo,* we utilized mice with a cardiomyocyte-specific overexpression of the glucose transporter GLUT1; hearts from these mice, which have been characterized previously (Luptak et al. 2007), do not exhibit contractile dysfunction. Hearts isolated from transgenic mice fed the HC/LF diet exhibit significantly lower *mhcα* expression, with a concomitant greater *mhcβ* expression, compared to hearts isolated from wild-type littermates, fed the same diet (Figs. 2A–2B). Feeding mice the LC/HF diet prevented this isoform switching; *mhcα* expression was not significantly different between wild-type and transgenic mice fed the LC/HF diet, whereas *mhcβ* was significantly lower in hearts isolated from transgenic versus wild-type mice fed the LC/HF diet (Fig. 2B). As such, *mhc* isoform switching (*mhcα*/*mhcβ* ratio) observed for hearts isolated from transgenic mice was prevented when mice were fed the LC/HF diet (Fig. 2C).

### Limiting dietary carbohydrate availability prevents *mhc* isoform switching in response to pressure overload

We have shown before that nine days of aortic constriction induces left ventricular hypertrophy in rats fed either HC/LF or LC/HF diet (i.e. diet-independent); diet alone had no effects on body weight, or heart weight (Young et al. 2001b). The same was the case in the present study (Table 1).

**Figure 3:**
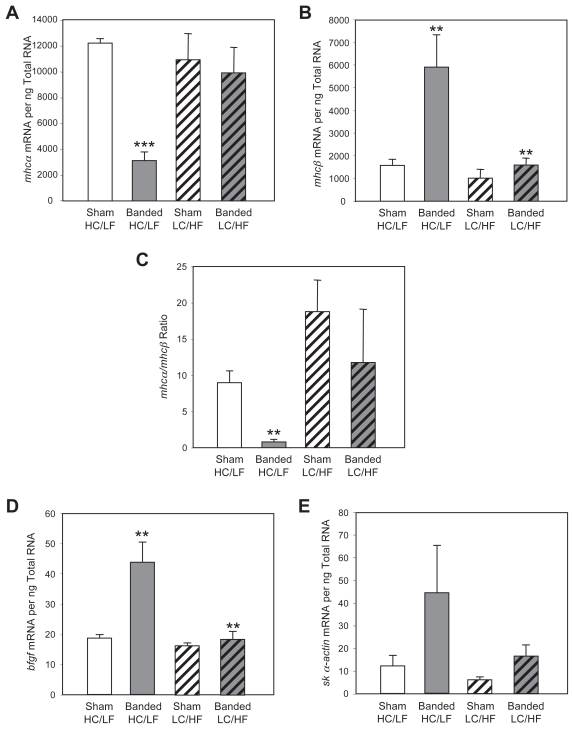
Effects of altered dietary carbohydrate and fat availability on *mhcα* (**A**) and *mhcβ* (**B**) expression, the *mhcα*/*mhcβ* ratio (**C**), as well as *bfgf* (**D**) and *sk α-actin* (**E**) expression, in sham and banded (aortic constriction) hearts. Rats were placed on either a high carbohydrate/low fat diet (HC/LF) or a low carbohydrate/high fat diet (LC/HF). Values are shown as the mean ± S.E.M. for five to ten separate observations. *, p < 0.05; **, p < 0.01; and ***, p < 0.001 *versus* HC/LF sham.

Aortic constriction of animals placed on the HC/LF diet resulted in decreased *mhcα* mRNA levels (Fig. 3A), and a concomitant increase in *mhcβ* mRNA levels (Fig. 3B). This isoform switching was not observed in response to pressure overload when rats were placed on the LC/HF diet (Figs. 3A–3B). Diet alone had no effects on MHC isoform expression in the heart (Figs. 3A–3B, black columns). As such, pressure overload-induced *mhc* isoform switching (as indicated by the *mhcα*/*mhcβ* ratio) was prevented when rats were fed the LC/HF diet (Fig. 3C). We also measured transcript levels of the “fetal” genes basic fibroblast growth factor (*bfgf*) and skeletal α-actin (*sk α-actin*) (Figs. 3D–3E). Like the transcript levels of MHCβ, pressure overload-mediated induction of these two transcripts was attenuated when rats were fed the LC/HF diet.

### Sustained pressure overload activates the hexosamine biosynthetic pathway

To investigate whether increased flux through the hexosamine biosynthetic pathway occurs in response to pressure overload in the heart, we measured: 1) the expression of two isoforms of GFAT (glutamine fructose 6-phosphate amido-transferase, *gfat1* and *gfat2*) which catalyzes the flux generating step in this pathway; and 2) UDP-N-acetylglucosamine, the principal end-product of this pathway, in control as well as in hypertrophied hearts (Veerababu et al. 2000; Yki-Jarvinen et al. 1998; Rossetti et al. 1995).

**Figure 4:**
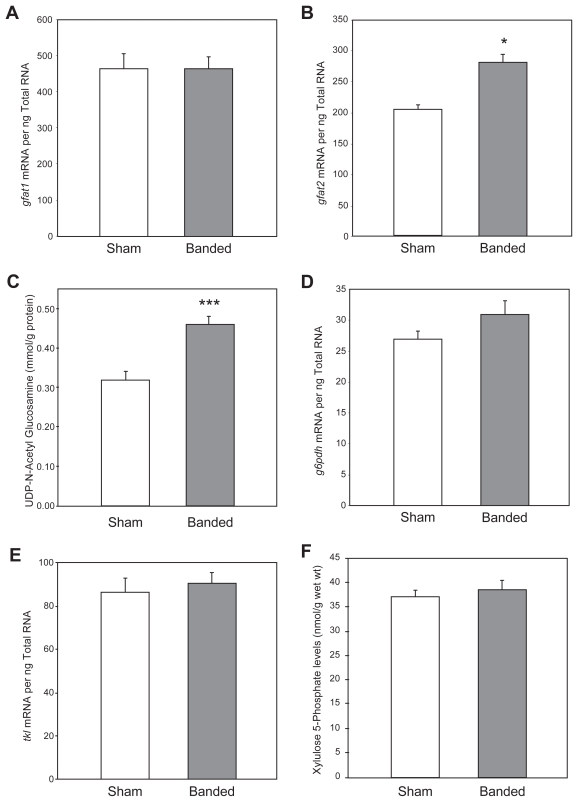
Effects of pressure overload on *gfat1* mRNA (**A**), *gfat2* mRNA (**B**) and UDP N-acetyl glucosamine (**C**), *g6pdh* mRNA (**D**), *tkl* mRNA (**E**), and xylulose 5-phosphate (**F**) levels in the heart. Values are shown as the mean ± S.E.M. for six to twenty-four separate observations. *, p < 0.05 and ***, p < 0.001.

Ascending aortic constriction for seven weeks induced hypertrophy (heart-to-body weight ratios 2.84 ± 0.02 vs 3.69 ± 0.05, control vs banded; p < 0.001). The level of *gfat1* expression was not significantly different between the control and hypertrophic hearts (Fig. 4A). In contrast, the level of *gfat2* mRNA was 38% higher in pressure overloaded hearts compared to control hearts (Fig. 4B). In addition, pressure overload was also associated with a 40% increase in cardiac UDP-N-acetylglucosamine levels (Fig. 4C).

### No effect of sustained pressure overload on the pentose phosphate pathway

To investigate whether increased flux through the pentose phosphate pathway occurs in response to pressure overload in the heart, seven weeks after aortic constriction we measured in control and hypertrophied hearts: 1) the expression of two key enzymes in xylulose 5-phosphate biosynthesis, namely *g6pdh* (glucose 6-phosphate dehydrogenase) and *tkl* (transketolase); and 2) xylulose 5-phosphate, an important regulatory intermediate in this pathway. The results indicate that neither the expression of *g6pdh* nor *tkl* were affected by sustained pressure overload (Figs. 4D–4E). Xylulose 5-phosphate levels were also not different (Fig. 4F).

## Discussion

**Figure 5:**
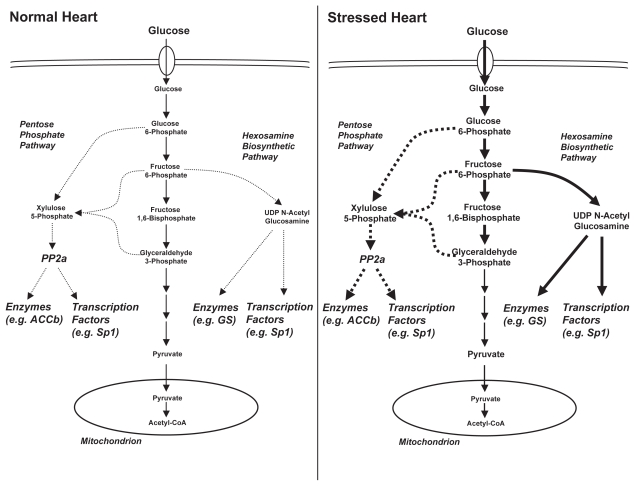
Hypothetical mechanism of glucose sensing in the heart in response to hemodynamic or metabolic stress. A loss of synchronization between glucose influx into the cardiomyocyte and pyruvate oxidation during pressure overload results in accumulation of glyoclytic intermediates. The latter can cause an increased flux through the pentose phosphate pathway (via G6PDH or TKL) and the hexosamine biosynthethic pathway (via GFAT), resulting in accumulation in xylulose 5-phosphate and UDP N-acetyl glucosamine, respectively. These metabolites affect the activity of various enzymes and transcription factors for phosphorylation and/or glycosylation events.

We provide strong evidence in support of the hypothesis that glucose metabolites play a role in the adaptation of the myocardium to pressure overload, at the level of gene expression. When hearts are forced to utilize glucose, both *ex vivo* (glucose perfused hearts) and *in vivo* (GLUT1 transgenic mice), *mhc* isoform switching is induced. Limiting dietary glucose prevents *mhc* isoform switching in both GLUT1 transgenic hearts and pressure overload-induced hypertrophied hearts. For potential mechanisms we focused on the hexosamine biosynthetic pathway and the pentose phosphate pathway (Fig. 5). Myocardial levels of *gfat2* mRNA (encoding for a flux generating enzyme in the hexosamine biosynthetic pathway) and UDP-N-acetylglucosamine (the major end-product of this pathway) were elevated in response to pressure overload. In contrast, no alterations in the expression of two key enzymes in the pentose phosphate pathway (namely *g6pdh* and *tkl*) were observed in the hypertrophied heart, nor was there an increase in xylulose 5-phosphate levels.

### Glucose sensing in the heart

A hallmark of long-term adaptation of the rodent heart to environmental stresses is the re-expression of a fetal gene program. The predominant isoform of myosin heavy chain (*mhc*) expressed in the fetal heart of the rodent is *mhcβ*. During the first weeks of post-natal life, *mhcβ* declines and is replaced by the adult isoform, *mhcα* (Schwartz et al. 1992). In the presence of chronic stimuli, including pressure overload, mechanical unloading or diabetes, the heart re-expresses *mhcβ* with concomitant repression of *mhcα* expression (Depre et al. 1998; Mercadier et al. 1981). To date, no single pathway has been attributed to this isoform switching in the heart to these various stimuli.

We propose a unifying hypothesis that is based on the following reasoning. Despite the apparent opposing metabolic adaptation of the heart to alterations in workload and a diabetic milieu, we propose that glycolytic intermediates accumulate in the heart during both situations due to an imbalance between glucose uptake and phosphorylation on the one hand and pyruvate oxidation on the other hand (Fig. 5). In the hypertrophied heart (and the atrophied and fetal hearts), glucose uptake and metabolism are both increased (Allard et al. 1994; Doenst et al. 2001). However, the high rate of glucose uptake exceeds the rate of pyruvate oxidation, resulting in accumulation of glycolytic intermediates. A similar scenario may occur for the heart during hypoxia, where decreased expression of peroxisome proliferator-activated receptor α-(PPARα-) regulated genes is associated with increased reliance on glucose (Razeghi et al. 2001). We designed the experiments with the assumption that with LC/HF we significantly reduced carbohydrate availability, although neither blood glucose nor insulin levels were measured. Previously published studies have shown lower plasma glucose and insulin levels in the fed state for rats fed a high fat (low carbohydrate) diet (De Gasquet et al. 1977). This pattern is markedly different from insulin-dependent diabetes mellitus, where both glucose and fatty acids levels are markedly elevated. In contrast to the hypertrophied heart, the diabetic heart increases reliance on fatty acids as a fuel. Fatty acids inhibit glucose oxidation at the level of the pyruvate dehydrogenase complex (PDC) to a greater extent than they inhibit glucose uptake. Increased mitochondrial acetyl-CoA and NADH levels (due to increased fatty acid and ketone body utilization) and phosphorylation by PDC kinase 4 (due to PPARα-mediated induction) jointly inhibit PDC (Wu et al. 1999; Randle et al. 1978). Despite decreased glucose transporter expression and decreased insulin-mediated glucose transport, the rates of glucose uptake in the diabetic environment, are comparable to those observed in normal hearts, due to the prevailing hyperglycemia (Ungar et al. 1955). Thus, with normal glucose influx into the cardiomyocyte, and the block at PDC, glucose metabolites accumulate. Indeed, intracellular concentrations of glucose, glucose 6-phosphate, fructose 6-phosphate, glycogen, pyruvate and lactate have all been shown to be increased in the heart during diabetes (Chen et al. 1984; Stroedter et al. 1995).

Additional evidence exists in support of the hypothesis. If the rate of glucose oxidation is increased in the pressure overloaded heart, thereby re-coupling glycolysis and pyruvate oxidation, through treatment of rats with etomoxir, an irreversible inhibitor of carnitine palmitoyltransferase I, *mhc* isoform switching is attenuated (Turcani and Rupp, 1997). Similar results are observed for the heart in diabetes. Myocardial *mhc* isoform switching during diabetes is prevented by treatment with etomoxir, as well as a second fatty acid oxidation inhibitor, methylpalmoxirate (Dillmann, 1985; Rupp et al. 1994). It therefore appears that whenever there is a loss of coupling between glycolysis and pyruvate oxidation, either due to increased glucose influx (e.g. fetal, hypertrophied and atrophied hearts) and/or inhibition of pyruvate oxidation (e.g. heart in diabetes), *mhc* isoform switching occurs. Glucose sensing may be the common mechanism. In addition to the *mhc* isoforms, the expression of various other genes is altered in the above situations. For example, skeletal α-actin is induced during pressure overload, an effect that is abolished when substrate switching is prevented (Young et al. 2001a) (Fig. 3E). Furthermore, induction of skeletal α-actin during pressure overload is dependent on the activation of Sp1, a transcription factor that is known to be activated in response to increased glucose availability in liver (Karns et al. 1995). More recently the nuclear receptor LXR has been identified as a glucose sensor in the lever as well (Mitro et al. 2007). In this tissue, LXR serves as a switch for the integration of various metabolic pathways.

While fatty acids are able to modulate gene expression in the heart, most likely through activation of the nuclear receptor PPARα, (Barger and Kelly, 2000) nothing is known concerning glucose-regulated gene expression in the heart. Extensive work in liver suggests the involvement of two major pathways in the sensing of glucose availability, namely the hexosamine biosynthetic pathway and the pentose phosphate pathway (Girard et al. 1997; Ferré 1999; Vaulont et al. 2000). The present studies provide evidence that glucose sensing occurs in the heart.

### Hexosamine biosynthetic pathway

Increased influx of glucose into the cell, and/or increased activity of GFAT, results in increased flux through the hexosamine biosynthetic pathway (Hebert, Jr., et al. 1996). The resultant elevation in the principal end-product of this pathway, UDP-N-acetylglucosamine, is utilized by transferases for the O-linked glycosylation of various target proteins (Wells et al. 2001). Such O-linked glycosylation of signaling proteins occurs at serine and threonine residues otherwise utilized as regulatory phosphorylation sites, a mechanism similar to phosphorylation in that it is a reversible covalent modification that affects the activity of the target protein (Wells et al. 2001). However, unlike phosphorylation, the rate of O-linked glycosylation is dependent on intracellular levels of the linkage group donor (i.e. UDP-N-acetylglucosamine, which in turn is dependent upon the influx of glucose into the cell, and/or increased activity of GFAT).

Proteins known to be activated by O-linked glycosylation include the transcription factors c-myc and Sp1, both of which are activated during pressure overload (Karns et al. 1995; Wells et al. 2001; Izumo et al. 1988). O-glycosylation of Sp1 is already known for quite some time (Jackson and Tjian, 1988). The present study shows that UDP-N-acetylglucosamine levels were significantly elevated in pressure overloaded hearts (Fig. 4C). It is reasonable to assume that this increase is due to both increased expression of *gfat2* (Fig. 4B), as well as increased influx of glucose into the cell. We speculate that c-myc and/or Sp1 activation in the heart during pressure overload may be due, at least in part, to increased O-linked glycosylation. Thus, altered glucose metabolism would contribute toward early molecular events involved in remodeling of the heart during pressure overload and the return to the fetal gene program (Sugden and Clerk, 1998; Komuro and Yazaki, 1993; Depre et al. 1998; Mercardier et al. 1981; Dillmann, 1980; Depre et al. 2000).

### Pentose phosphate pathway

We have previously shown appreciable flux through the non-oxidative branch of the PPP in the heart (Goodwin et al. 2001). However, neither the expression of key enzymes involved in this pathway (*g6pdh* and *tkl*), nor the levels of xylulose 5-phosphate, were altered in the heart after seven weeks of pressure overload (Figs. 4D–4F). This suggests that the pentose phosphate pathway may not play a major role in adaptation of the heart to sustained pressure overload. Still, this pathway may be important for acute adaptation to stimuli such as ischemia, when glycolytic intermediates accumulate rapidly. It is certainly important in the liver, where the signaling molecule xylulose 5-phospthate triggers rapid changes in the nuclear import of the transcription factor carbohydrate response element binding protein (ChREBP) which coordinates the transcriptional regulation of metabolic enzymes (Uyeda and Repa, 2006). The role of ChREBP in the heart remains unknown.

## Conclusions

We have found evidence in support of the hypothesis that alterations in metabolism are involved in the adaptation of the heart to environmental stresses, such as pressure overload, at least at the level of gene expression. Increased O-linked glycosylation of proteins may play significant roles in the adaptation of the heart, in addition to transcriptional alterations, such as translational initiation (through eIF-2) or post-translational modulation of enzymatic activity (e.g. glycogen synthase) or cellular signaling (e.g. insulin signaling) (Datta et al. 1989; Crook et al. 1995; Patti et al. 1999). Glucose sensing may therefore play a role in the adaptation of the myocardium to various stimuli, whether they are hemodynamic (e.g. increased workload) or metabolic (e.g. diabetes).

## Figures and Tables

**Table 1:** Effects of banding and altered dietary fat content on body weight (BW), heart weight (HW), hypertrophy (HW/BW ratio).

	HC/LF Sham	HC/LF Banded	LC/HF Sham	LC/HF Banded
Body Weight (g)	255 ± 7	257 ± 6	267 ± 6	262 ± 6
Heart Weight (g)	0.81 ± 0.03	0.99 ± 0.09*	0.85 ± 0.02	1.02 ± 0.07†
HW/BW (×100)	3.16 ± 0.08	3.91 ± 0.24*	3.17 ± 0.07	3.90 ± 0.13†

Values are shown as the mean ± S.E.M. for between five and ten separate observations.

* , p < 0.05 *versus* HC/LF sham.

† , p < 0.05 *versus* LC/HF banded.

**Table 2:** Primer and probe sequences used in real time quantitative RT-PCR.

Gene	Primer/Probe	Sequence
*mhcα*	Forward	5′-TGAAAAGATTAACCGGAGTTTAAGA-3′
	Reverse	5′-CAGGCACGAAGCACTCTGTG-3′
	Probe	5′-FAM-CCIAAGTCAGCCATCTGGGCATC-TAMRA-3′
*mhcβ*	Forward	5′-TCCTCCCTCAAGCTCCTAAGTAA-3′
	Reverse	5′-TTTGCCTTTGCCCTTGTCTA-3′
	Probe	5′-FAM-CATCAGCICCAGCATAGTTGGCAAACA-TAMRA-3′
*gfat1*	Forward	5′-GCCGAGCTGTGCAAACTCT-3′
	Reverse	5′-GGCTGCTCAAAAATTTCCTTC-3′
	Probe	5-′FAM-CTCCAGCAGATCATGAAGGGCAACTTTAGT-TAMRA-3′
*gfat2*	Forward	5′-GCCAGTTCATCTCTCTGGTGC-3′
	Reverse	5′-ATCTGAGGCCACGGATGAT-3′
	Probe	5′-FAM-TGGTTTGATGATGTCTGAAGATCGAATTTCTC-TAMRA-3′
*g6pdh*	Forward	5′-GGGCAAAGAGATGGTCCAG-3′
	Reverse	5′-TCGATTCCAGATGGGTCCA-3′
	Probe	5′-FAM-AGATCCTGTTGGCAAATCTCAGCACCA-TAMRA-3′
*tkl*	Forward	5′-CGAAACCCTCACAATGATCG-3′
	Reverse	5′-AGCTTCAGCCCAGACTGCA-3′
	Probe	5′-FAM-TTTGTGCTCTCCAAGGGCCATGC-TAMRA-3′

## References

[b1-grsb-2007-251] Allard MF Schonekess BO Henning SL 1994 Contribution of oxidative metabolism and glycolysis to ATP production in hypertrophied hearts Am J Physiol 267 H742 H50 8067430 10.1152/ajpheart.1994.267.2.H742

[b2-grsb-2007-251] Barger PM Kelly DP 2000 PPAR signaling in the control of cardiac energy metabolism Trends Cardiovasc Med 10 238 45 11282301 10.1016/s1050-1738(00)00077-3

[b3-grsb-2007-251] Casazza JP Veech RL 1986 The measurement of xylulose 5-phosphate, ribulose 5-phosphate, and combined sedoheptulose 7-phosphate and ribose 5-phosphate in liver tissue Anal Biochem 159 243 8 3826613 10.1016/0003-2697(86)90338-6

[b4-grsb-2007-251] Chen V Ianuzzo C Fong B 1984 The effects of acute and chronic diabetes on myocardial metabolism in rats Diabetes 33 1078 84 6500187 10.2337/diab.33.11.1078

[b5-grsb-2007-251] Chomczynski P Sacchi N 1987 Single-step method of RNA isolation by acid guanidium thiocyanate-phenol-chloroform extraction Anal Biochem 162 159 69 10.1006/abio.1987.9999 2440339

[b6-grsb-2007-251] Crook ED Zhou J Daniels M 1995 Regulation of glycogen synthase by glucose, glucosamine, and glutamine:fructose-6-phosphate amidotransferase Diabetes 44 314 20 7883119 10.2337/diab.44.3.314

[b7-grsb-2007-251] Datta B Ray MK Chakrabarti D 1989 Glycosylation of eukaryotic peptide chain initiation factor 2 (eIF-2)-associated 67-kDa polypeptide (p67) and its possible role in the inhibition of eIF-2 kinase-catalyzed phosphorylation of the eIF-2 alpha-subunit J Biol Chem 264 20620 4 2511207

[b8-grsb-2007-251] De Gasquet P Griglio S Pequignot-Planche E 1977 Diurnal changes in plasma and liver lipids and lipoprotein lipase activity in heart and adipose tissue in rats fed a high and low fat diet J Nutr 107 199 212 556762 10.1093/jn/107.2.199

[b9-grsb-2007-251] Depre C Shipley GL Chen W 1998 Unloaded heart in vivo replicates fetal gene expression of cardiac hypertrophy Nat Med 4 1269 75 9809550 10.1038/3253

[b10-grsb-2007-251] Depre C Young ME Ying J 2000 Streptozotocin-induced changes in cardiac gene expression in the absence of severe contractile dysfunction J Mol Cell Cardiol 32 985 96 10888252 10.1006/jmcc.2000.1139

[b11-grsb-2007-251] Dillmann WH 1980 Diabetes mellitus induces changes in cardiac myosin of the rat Diabetes 29 579 82 6445843 10.2337/diab.29.7.579

[b12-grsb-2007-251] Dillmann WH 1985 Methyl palmoxirate increases Ca^2+^-myosin ATPase activity and changes myosin isoenzyme distribution in the diabetic rat heart Am J Physiol 248 E602 E6 3158215 10.1152/ajpendo.1985.248.5.E602

[b13-grsb-2007-251] Doenst T Goodwin GW Cedars AM 2001 Load-induced changes in vivo alter substrate fluxes and insulin responsiveness of rat heart in vitro Metabolism 50 1083 90 11555843 10.1053/meta.2001.25605

[b14-grsb-2007-251] Ferré P 1999 Regulation of gene expression by glucose Proc Nutr Soc 58 621 3 10604195 10.1017/s0029665199000816

[b15-grsb-2007-251] Gibson UEM Heid CA Williams PM 1996 A novel method for real time quantitative RT-PCR Genome Res 6 995 1001 8908519 10.1101/gr.6.10.995

[b16-grsb-2007-251] Girard J Ferre P Foufelle F 1997 Mechanisms by which carbohydrates regulate expression of genes for glycolytic and lipogenic enzymes Annu Rev Nutr 17 325 52 9240931 10.1146/annurev.nutr.17.1.325

[b17-grsb-2007-251] Goodwin GW Cohen DM Taegtmeyer H 2001 [5-3H]glucose overestimates glycolytic flux in isolated working rat heart: role of the pentose phosphate pathway Am J Physiol Endocrinol Metab 280 E502 E8 11171606 10.1152/ajpendo.2001.280.3.E502

[b18-grsb-2007-251] Goodwin GW Taylor CS Taegtmeyer H 1998 Regulation of energy metabolism of the heart during acute increase in heart work J Biol Chem 273 29530 9 9792661 10.1074/jbc.273.45.29530

[b19-grsb-2007-251] Hebert LF Jr Daniels MC Zhou J 1996 Overexpression of glutamine:fructose-6-phosphate amidotransferase in transgenic mice leads to insulin resistance J Clin Invest 98 930 6 8770864 10.1172/JCI118876 PMC507507

[b20-grsb-2007-251] Izumo S Nadal-Ginard B Mahdavi V 1988 Protooncogene induction and reprogramming of cardiac gene expression produced by pressure overload Proc Natl Acad Sci USA 85 339 43 2963328 10.1073/pnas.85.2.339 PMC279543

[b21-grsb-2007-251] Jackson SP Tjian R 1988 O-glycosylation of eukaryotic transcription factors: implications for mechanisms of transcriptional regulation Cell 55 125 33 3139301 10.1016/0092-8674(88)90015-3

[b22-grsb-2007-251] Karns L Kariya K Simpson P 1995 M-CAT, CArG, and Sp1 elements are required for alpha 1-adrenergic induction of the skeletal alpha-actin promoter during cardiac myocyte hypertrophy. Transcriptional enhancer factor-1 and protein kinase C as conserved transducers of the fetal program in cardiac growth J Biol Chem 270 410 7 7814403 10.1074/jbc.270.1.410

[b23-grsb-2007-251] Kleinman L Wechsler A Rembert J 1978 A reproducible model of moderate to severe concentric left ventricular hypertrophy Am J Physiol 234 H515 H9 148215 10.1152/ajpheart.1978.234.5.H515

[b24-grsb-2007-251] Komuro I Yazaki Y 1993 Control of cardiac gene expression by mechanical stress Annu Rev Physiol 55 55 75 8466185 10.1146/annurev.ph.55.030193.000415

[b25-grsb-2007-251] Luptak I Yan J Curi L 2007 Long-term effects of increased glucose entry on mouse hearts during normal aging and ischemic stress Circulation 116 901 9 17679614 10.1161/CIRCULATIONAHA.107.691253

[b26-grsb-2007-251] Mercadier JJ Lompre AM Wisnewsky C 1981 Myosin isoenzymic changes in several models of rat cardiac hypertrophy Circ Res 49 525 32 6454511 10.1161/01.res.49.2.525

[b27-grsb-2007-251] Mitro N Mak PA Vargas L 2007 The nuclear receptor LXR is a glucose sensor Nature 445 219 23 17187055 10.1038/nature05449

[b28-grsb-2007-251] Patti ME Virkamaki A Landaker EJ 1999 Activation of the hexosamine pathway by glucosamine in vivo induces insulin resistance of early postreceptor insulin signaling events in skeletal muscle Diabetes 48 1562 71 10426374 10.2337/diabetes.48.8.1562

[b29-grsb-2007-251] Randle P Sugden P Kerbey A 1978 Regulation of pyruvate oxidation and the conservation of glucose Biochem Soc Symp 43 47 67 373769

[b30-grsb-2007-251] Razeghi P Young ME Abbasi S 2001 Hypoxia in vivo decreases peroxisome proliferator-activated receptor alpha-regulated gene expression in rat heart Biochem Biophys Res Commun 287 5 10 11549245 10.1006/bbrc.2001.5541

[b31-grsb-2007-251] Rossetti L Hawkins M Chen W 1995 In vivo glucosamine infusion induces insulin resistance in normoglycemic but not in hyperglycemic conscious rats J Clin Invest 96 132 40 7615783 10.1172/JCI118013 PMC185181

[b32-grsb-2007-251] Rupp H Elimban V Dhalla NS 1994 Modification of myosin isozymes and SR Ca(2+)-pump ATPase of the diabetic rat heart by lipid-lowering interventions Mol Cell Biochem 132 69 80 8078510 10.1007/BF00925676

[b33-grsb-2007-251] Schwartz K Carrier L Chassagne C 1992 Regulation of myosin heavy chain and actin isogenes during cardiac growth and hypertrophy Symp Soc Exp Biol 46 265 72 1341040

[b34-grsb-2007-251] Stroedter D Schmidt T Bretzel R 1995 Glucose metabolism and left ventricular dysfunction are normalized by insulin and islet transplantation in mild diabetes in the rat Acta Diabetol 32 235 43 8750762 10.1007/BF00576256

[b35-grsb-2007-251] Sugden PH Clerk A 1998 Cellular mechanisms of cardiac hypertrophy J Mol Med 76 725 46 9826118 10.1007/s001090050275

[b36-grsb-2007-251] Taegtmeyer H Hems R Krebs HA 1980 Utilization of energy-providing substrates in the isolated working rat heart Biochem J 186 701 11 6994712 10.1042/bj1860701 PMC1161705

[b37-grsb-2007-251] Turcani M Rupp H 1997 Etomoxir improves left ventricular performance of pressure-overloaded rat heart Circulation 96 3681 6 9396471 10.1161/01.cir.96.10.3681

[b38-grsb-2007-251] Ungar I Gilbert M Siegel A 1955 Studies on myocardial metabolism: IV. Myocardial metabolism in diabetes Am J Med 18 385 96 14349963 10.1016/0002-9343(55)90218-7

[b39-grsb-2007-251] Uyeda K Repa JJ 2006 Carbohydrate response element binding protein, ChREBP, a transcription factor coupling hepatic glucose utilization and lipid synthesis Cell Metab 4 107 10 16890538 10.1016/j.cmet.2006.06.008

[b40-grsb-2007-251] Vaulont S Vasseur-Cognet M Kahn A 2000 Glucose regulation of gene transcription J Biol Chem 275 31555 8 10934218 10.1074/jbc.R000016200

[b41-grsb-2007-251] Veerababu G Tang J Hoffman RT 2000 Overexpression of glutamine: fructose-6-phosphate amidotransferase in the liver of transgenic mice results in enhanced glycogen storage, hyperlipidemia, obesity, and impaired glucose tolerance Diabetes 49 2070 8 11118009 10.2337/diabetes.49.12.2070

[b42-grsb-2007-251] Wells L Vosseller K Hart GW 2001 Glycosylation of nucleocytoplasmic proteins: signal transduction and O-GlcNAc Science 291 2376 8 11269319 10.1126/science.1058714

[b43-grsb-2007-251] Wu P Inskeep K Bowker-Kinley MM 1999 Mechanism responsible for inactivation of skeletal muscle pyruvate dehydrogenase complex in starvation and diabetes Diabetes 48 1593 9 10426378 10.2337/diabetes.48.8.1593

[b44-grsb-2007-251] Yki-Jarvinen H Virkamaki A Daniels MC 1998 Insulin and glucosamine infusions increase O-linked N-acetyl-glucosamine in skeletal muscle proteins in vivo Metabolism 47 449 55 9550544 10.1016/s0026-0495(98)90058-0

[b45-grsb-2007-251] Young ME Laws FA Goodwin GW 2001a Reactivation of peroxisome proliferator-activated receptor alpha is associated with contractile dysfunction in hypertrophied rat heart J Biol Chem 276 44390 5 11574533 10.1074/jbc.M103826200

[b46-grsb-2007-251] Young ME Patil S Ying J 2001b Uncoupling protein 3 transcription is regulated by peroxisome proliferator-activated receptor (alpha) in the adult rodent heart FASEB J 15 833 45 11259402 10.1096/fj.00-0351com

